# Isolated renal nocardiosis in a patient with AIDS: Unusual presentation

**DOI:** 10.4103/0970-1591.56178

**Published:** 2009

**Authors:** K. Pai, L. Rao

**Affiliations:** Department of Pathology, Karturba Medical College, Manipal, Karnataka, India

**Keywords:** Immunocompromised, nocardiosis, renal

## Abstract

Disseminated or systemic infection with nocardiosis is an opportunistic infection that is seen in immunocompromised individuals and can involve any organ. The primary infection in systemic nocardiosis usually occurs in the lungs and subsequently hematogenous dissemination occurs in other organs of the body. Nocardia infection of the kidney usually manifests as multiple pyelonephritic abscesses. We report a case of isolated renal nocardiosis, without involvement of the lungs or other organs in a patient with AIDS who presented with symptoms of renal failure. The nephrectomy specimen showed multiple calculi in the calyceal system and a tumorous mass with necrotic areas, which histologically showed features of nocardiosis. The case is being presented as this is an unusual manifestation of renal nocardiosis.

## INTRODUCTION

Systemic nocardiosis is a serious infectious disease characterized by infection of the brain or two nonadjacent organs. Isolated genitourinary involvement is rare and involvement of the renal parenchyma usually occurs during the aggressive course of acute systemic nocardiosis.[1]

There are very few reports describing involvement of the kidneys in literature that describe the lesions to manifest as multiple pyelonephritic abcesses. We report a case of renal nocardiosis in a patient with AIDS, which occurred in the absence of involvement of any other organs. A nephrectomy was performed for a non functioning kidney due to hydronephrosis secondary to urolithiasis and pyonephrosis. The case showed an unusual pathological feature of a large tumorous mass replacing most of the renal parenchyma, which showed histological evidence of nocardiosis infection.

## CASE REPORT

A 55-year-old male presented with symptoms of pain in the left loin for 4 months, intermittent fever for 1 year, and easy fatigability for 2 years. The pain in the abdomen was intermittent, crampy, and was associated with burning micturition. Two years earlier, the patient was evaluated for easy fatigability and weakness and was diagnosed to be seropositive for HIV. He was put on antiretroviral therapy, which he discontinued on his own after 1 month.

Upon investigation, he was found to be anemic (7.1 g/dl). His white blood cell (WBC) count was in the normal range, but showed toxic granules. His erythrocyte sedimentation rate was high (140 mm/1 hr).

A urine microscopy showed the presence of numerous pus cells, granular casts, and few red blood cells (RBC). Blood urea and creatinine were elevated (55 mg/dl and 2.4 mg/dl, respectively). An ultrasonography showed a dilated pelvicalyceal system with calculi and large low amplitude moving echogenic area suggestive of pyonephrosis in a hydronephrotic kidney with urolithiasis. Computed tomography also suggested a diagnosis of hydronephrosis with pyonephrosis. A clinical diagnosis of urolithiasis with hydronephrosis and pyonephrosis was made and a nephro-ureterectomy was performed for the non functioning kidney and to eliminate the source of infection as the patient had recurrent episodes of fevers in the past. Pre-operatively, involvement of the adjoining organs or enlarged nodes was not seen.

### Pathology

**Figure 1:**
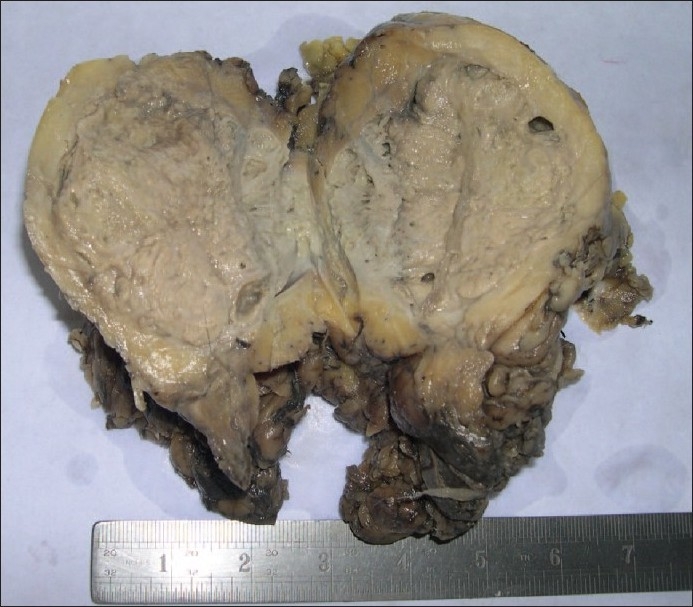
A nephrectomy specimen showing a large tumorous mass replacing the entire kidney

**Figure 2:**
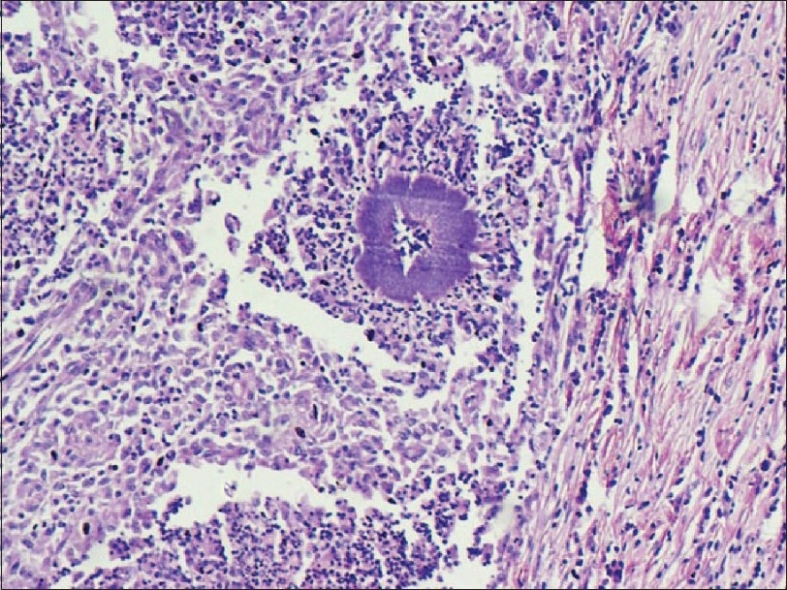
A microscopic examination shows basophilic colonies surrounded by neutophilic abscesses and granulation tissue, (H&E, 100X)

The gross specimen of nephrectomy showed a large tumorous mass measuring 9 × 6 cm replacing the entire kidney with grayish-yellow areas and necrotic areas. Normal renal parenchyma could not be identified. Many cystic spaces with impacted calculi were seen [Figure 1]. A microscopic examination showed multiple basophilic colonies surrounded by neutrophilic abscesses and granulation tissue [Figure 2] replacing most of the renal parenchyma, which showed features of chronic pyelonephritis. Special stains to differentiate from actinomycosis were done, which revealed that the colonies stained weakly positive with methanamine silver and stained positive with acid fast stain. A diagnosis of renal nocardiosis with chronic pyelonephritis was made.

A detailed evaluation of the patient was subsequently made to rule out the involvement of other systems. A chest X-ray was normal. No other symptoms or signs pertaining to any other organ involvement were found. Following the histological diagnosis, the patient was treated with broad spectrum systemic anti-fungal and anti-retroviral treatment.

## DISCUSSION

Nocardiosis is an infection caused by the bacterium of the genus nocardia, most commonly nocardia asteroides or nocardia brasiliensis. Normally found in soil, these organisms cause occasional sporadic disease in humans. The usual mode of transmission is inhalation of organisms suspended in dust. Generally, nocardial infection requires some degree of immune suppression. It presents as acute, subacute, or chronic infectious disease that occurs in cutaneous, pulmonary, and disseminated forms. Disseminated nocardiosis may involve any organ; lesions in the brain or meninges are the most frequent.

Genitourinary involvement is rare and involvement of the renal parenchyma usually occurs during the aggressive course of acute systemic nocardiosis and usually presents as pyelonephritic microabcesses.[2,3]

The diagnosis of nocardiosis was made upon histological examination by the presence of basophilic colonies of fungi, which are surrounded by neutrophilic abscesses and granulation tissue. Differentiation from actinomycosis, which also has similar histological features, can be made with the help of acid fast stain. Fungal filaments of nocardiosis stains positive while actinomycosis is acid fast negative.

Most patients with disseminated nocardiosis have underlying immunocompromising disease or are receiving immunosuppressive therapy and the infection results from hematogenous dissemination, usually from a pulmonary focus. One-half of all cases of pulmonary nocardiosis also involve infections in areas outside the lungs, and approximately 20% of patients with disseminated disease present solely with extrapulmonary disease, which usually spreads hematogenously from an asymptomatic or healed pulmonary site. The kidney involvement in our case probably occurred from the spread of an asymptomatic or healed pulmonary site.[4] The damage caused to the kidney by stones with consequent hydonephrosis possibly predisposed to the infection to localize to the kidney by nocardia in our case.

Renal nocardiosis manifesting as a large tumorous mass is a rare presentation not described in literature to the best of our knowledge. Hence, this case is presented for its unusual presentation and to alert the clinician of the possibility of renal nocardiosis in an immunocompromised individual. However,, there is no finding on the CT scan or in the pre-operative investigation that is specific for this disease to make a pre-operative diagnosis of renal nocardiosis.
